# Predictive Modeling and Structure Analysis of Genetic Variants in Familial Hypercholesterolemia: Implications for Diagnosis and Protein Interaction Studies

**DOI:** 10.1007/s11883-023-01154-7

**Published:** 2023-10-17

**Authors:** Asier Larrea-Sebal, Shifa Jebari-Benslaiman, Unai Galicia-Garcia, Ane San Jose-Urteaga, Kepa B. Uribe, Asier Benito-Vicente, César Martín

**Affiliations:** 1 Department of Biochemistry and Molecular Biology, Universidad del País Vasco UPV/EHU, 48080 Bilbao, Spain; 2 Department of Molecular Biophysics, Biofisika Institute, University of Basque Country and Consejo Superior de Investigaciones Científicas (UPV/EHU, CSIC), 48940 Leioa, Spain; 3 Fundación Biofisika Bizkaia, 48940 Leioa, Spain

**Keywords:** Familial hypercholesterolemia, LDLR, APOB, PCSK9, Bioinformatics tools, Functional validation

## Abstract

**Purpose of Review:**

Familial hypercholesterolemia (FH) is a hereditary condition characterized by elevated levels of low-density lipoprotein cholesterol (LDL-C), which increases the risk of cardiovascular disease if left untreated. This review aims to discuss the role of bioinformatics tools in evaluating the pathogenicity of missense variants associated with FH. Specifically, it highlights the use of predictive models based on protein sequence, structure, evolutionary conservation, and other relevant features in identifying genetic variants within *LDLR*, *APOB*, and *PCSK9* genes that contribute to FH.

**Recent Findings:**

In recent years, various bioinformatics tools have emerged as valuable resources for analyzing missense variants in FH-related genes. Tools such as REVEL, Varity, and CADD use diverse computational approaches to predict the impact of genetic variants on protein function. These tools consider factors such as sequence conservation, structural alterations, and receptor binding to aid in interpreting the pathogenicity of identified missense variants. While these predictive models offer valuable insights, the accuracy of predictions can vary, especially for proteins with unique characteristics that might not be well represented in the databases used for training.

**Summary:**

This review emphasizes the significance of utilizing bioinformatics tools for assessing the pathogenicity of FH-associated missense variants. Despite their contributions, a definitive diagnosis of a genetic variant necessitates functional validation through in vitro characterization or cascade screening. This step ensures the precise identification of FH-related variants, leading to more accurate diagnoses. Integrating genetic data with reliable bioinformatics predictions and functional validation can enhance our understanding of the genetic basis of FH, enabling improved diagnosis, risk stratification, and personalized treatment for affected individuals. The comprehensive approach outlined in this review promises to advance the management of this inherited disorder, potentially leading to better health outcomes for those affected by FH.

## Introduction: Familial Hypercholesterolemia

Familial hypercholesterolemia (FH) is a common inherited autosomal semi-dominant disorder primarily characterized by high plasma levels of low-density lipoprotein cholesterol (LDL-C) due to impaired metabolism [1]. If left untreated, persistent elevation of LDL-C throughout a person’s lifetime can lead to the development of atherosclerotic plaques and an increased risk of premature cardiovascular disease [2]. The major genetic determinants of FH correspond to pathogenic variants in 3 genes that cover the 99% of FH cases: *LDLR*, *APOB* (apolipoprotein B), and *PCSK9* (Pro-protein Convertase Subtilisin/Kexin Type 9) [3]. The prevalence of FH in its heterozygous form (HeFH) has traditionally been estimated to be around 1 in 500 individuals. However, the frequency can vary between 1 in 200 and 1 in 300 depending on the specific criteria used to define FH (such as genetic variants, LDL-C threshold, clinical score, or a combination of factors) and the populations under study [4]. In the case of the homozygous form of the disease (HoFH), the prevalence has traditionally been estimated at 1 in 1 million individuals. However, recent studies have revealed a higher prevalence, with estimates reaching as high as 1 in 300,000 individuals [4]. Despite its high prevalence, FH is still underdiagnosed, with less than 1% of the patients diagnosed in most countries [5]. Although there is a consensus on the criteria required to diagnose FH there are several clinical scoring systems that evaluate differently the consensus parameters [6, 7]. Among them, Dutch Lipid Clinic Network (DLCN)5 and Simon Broome Register (SBR) [8] are the most used ones. Most FH clinical algorithms consider lipid values (total cholesterol and LDL-C levels), the presence of physical stigmata (tendon xanthoma or corneal arcus), cascade screening and pathogenic DNA variants. Functional validation plays a crucial role in achieving a correct and early diagnosis of FH through genetic testing, which is considered the preferred method for FH diagnosis. However, the majority of FH variants lack functional characterization, requiring additional measures to complement genetic testing for an accurate and definitive diagnosis [9].

### LDLR

**Fig. 1:**
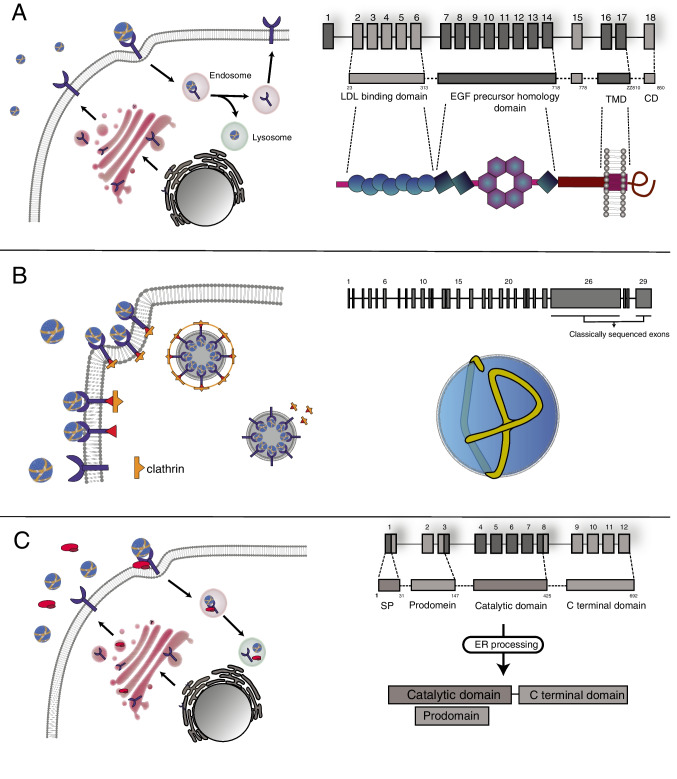
Cholesterol Homeostasis and Genetic Variants. (**A**) The *LDLR* gene encodes a transmembrane protein that regulates cholesterol homeostasis. LDLR has functional subdomains within its ectodomain and intracellular domain. It interacts with lipoproteins and PCSK9, leading to internalization and degradation. Pathogenic LDLR variants are classified into six subclasses based on their effects. (**B**) The *APOB* gene encodes apoB-100, a key component of lipoproteins and the primary ligand for LDLR. The conformation and binding regions of apoB-100 influence its affinity for LDLR. *APOB* pathogenic variants can occur outside these binding regions, making the identification of pathogenic variants more challenging. This suggests that conformational modifications of apoB-100 may play a significant role in its interaction with LDLR. (C) PCSK9 is synthesized as a zymogen and undergoes autocatalysis to release a peptide that inactivates its catalytic activity. Binding of PCSK9 to the LDLR-EGF domain prevents the conformational change required for receptor recycling. Upon endosome acidification, PCSK9’s affinity for LDLR increases, leading to the degradation of the LDLR-PCSK9 complex in the lysosome. GOF variants increase LDLR degradation, resulting in elevated LDL-C levels and increased risk of CVD

The *LDLR* gene located on 19p13.2 chromosome encodes a type I transmembrane protein of 839 amino acids, the LDLR, which regulates cholesterol homeostasis in mammalian cells [1] and constitutes the main gene associated with FH. Genetic variants in *LDLR* represent more than 90% of the FH causing variants, with more than 3000 variants annotated in ClinVar database [10]. LDLR is structured into functional subdomains organized within an ectodomain and intracellular domain (Fig. 1). The ectodomain contains the ligand binding domain (LBD), the epidermal growth factor precursor homology domain (EGF) and the O-linked domain. On the other hand, the intracellular domain harbors the transmembrane domain and the cytoplasmic domain, that targets the LDLR to clathrin-coated pits for the internalization of the LDLR-LDL complex [11, 12]. Binding of lipoproteins to the LDLR is mediated through the interaction of acidic residues in the LBD with basic residues of apoB-100 or ApoE [13]. Additionally, LDLR also interacts with PCSK9, a secreted protein that regulates membrane levels of LDLR through binding to EGF-LDLR domain, leading to degradation of LDLR in the endosome [14].

According to the region of the LDLR affected, the LDLR pathogenic variants can be classified into six sub-classes: class 1: LDLR is not synthesized, known as “null allele”; Class 2: LDLR is retained in the endoplasmic reticulum, completely or partially (2A and 2B, respectively); Class 3: Deficient binding to apoB-100; Class 4: Impaired endocytosis due to a deficient recruitment of the LDLR into clathrin-coated pits; Class 5: Impaired recycling; Class 6: impaired insertion in the membrane [3, 15]. LDLR pathogenic variants have been described along all domains and, depending on their location, they can affect the receptor function differently.

### APOB

The *APOB* gene located on the 2p24.1chromosome is a large and polymorphic gene spanning 43 kb in length, which constitutes the second most common cause of FH. The *APOB* gene comprises 29 exons and 28 introns, and encodes two forms of apolipoprotein B (apoB) in circulating lipoproteins, apoB-48 and apoB-100. ApoB-48 is produced by the small intestine, whereas full-length apoB-100 is produced in the liver. The mature form of apoB-100 is a protein of 4536 amino acids [16], which constitutes both the integral component of several lipoproteins (very low-density lipoprotein (VLDL) and LDL [17]) and the ligand for LDLR [18]. ApoB-100 interacts with lipids in a close manner, and the conformation that adopts the apolipoprotein within the lipid moiety confers the structure and physical properties to the lipoprotein [19] (Fig. 1). The apoB-100 domain that interacts with LDLR was first localized between residues 3386 and 3396 [20]. Although not proven experimentally, an eight-domain model for apoB-100 binding to LDLR was later proposed, in which the LDLR binding regions in apoB-100 expand between residues 2820–3202 and 3243–3498 [21]. The most frequent *APOB* pathogenic variant to date is p.R3527Q [22]. It has been shown that alterations in residue 3527 destabilize the protein, affecting the structure and, thus, the affinity for LDLR [23]. In addition to pathogenic variants within the putative binding domain, a growing number of pathogenic *APOB* variants are being described outside the putative binding regions, which complicates the identification of pathogenic variants [24]. In addition, this heterogeneity of pathogenic variants in *APOB* suggests that conformational modifications of the apolipoprotein could be a key player in the affinity of apoB-100 to LDLR [25].

### PCSK9

PCSK9 belongs to a family of 9 subtilisin-like serine proteases and plays a key function in plasma cholesterol metabolism by regulating LDLR levels through the promotion of LDLR degradation [26]. The *PCSK9* gene is located on the short arm of chromosome 1p32.3. PCSK9 is synthesized as a 72 kDa soluble zymogen (proPCSK9), which undergoes an autocatalytic process at the N-terminal domain. Upon autocatalysis, a 14 kDa peptide is released, which remains attached to the mature protein and inactivates the catalytic activity [27]. Upon binding to the LDLR-EGF domain, PCSK9 prevents the LDLR conformational change required to be recycled. This effect occurs after endosome acidification, which increases the affinity of PCSK9 for LDLR and leads the LDLR-PCSK9 complex to degradation in the lysosome [28] (Fig. 1). Large cohort studies have shown the existence of two major *PCSK9* variants, gain-of-function (GOF) and loss-of-function (LOF) [29, 30]. *PCSK9* pathogenic variants leading to GOF activity have been identified as the third genetic cause of FH [29, 31]. PCSK9 GOF variants increase LDLR degradation, resulting in higher circulating LDL-C levels, which directly increases the risk of developing CVD. On the other hand, LOF variants have a diminished effect on LDLR degradation, thus leading to lower LDL-C levels and decreased CVD risk. Both types of variants are broadly distributed along the three domains of the protein: the prodomain, the catalytic domain, and the C-terminal domain. The mechanisms by which the GOF/LOF PCSK9 variants affect LDLR degradation-rate are diverse, so predicting the effect of PCSK9 variants is complex [32].

#### Bioinformatics as a Clinical Tool

Functional characterization is a direct method to assess the activity of a variant by analyzing its effect on the biological processes in which the molecule is involved [15]. However, this is an arduous, time-consuming, and costly process that involves obtaining samples from the patient for subsequent purification or cloning the variant to further characterize their functionality in vitro. Additionally, during the past few years, high-throughput next-generation sequencing (NGS)-based methods have drastically increased the number of FH-related gene variants, opening the floodgates for the development of prediction models [33, 34]. On the other hand, the huge number of genetic variants being described through high-throughput NGS has increased to such an extent that it is almost impossible to functionally characterize all of them. Although functional characterization is essential for the proper analysis of a variant, in silico prediction tools offer a quick, cheap, and increasingly precise alternative.

Bioinformatic tools use the current knowledge about a protein or protein family to create in silico predictive models. These tools mostly rely on artificial intelligence (AI) to develop mathematical models capable of solving complex biological problems by analyzing vast datasets and intricate molecular interactions, ultimately aiding in the prediction of protein structure, function, and interactions [35]. AI involves the development of intelligent systems that act rationally in response to the given inputs. Machine learning (ML), one of the most well-known AI disciplines, applies statistical models and algorithms to analyze data. In contrast to classical programming, where known features (inputs) are used to create the algorithm, ML may use novel or different combinations of inputs and weights [36].

The most important parameters when developing a ML model are the dataset used in the training of the model and the approach used to optimize the results. Depending on the datasets used, an ML model can be supervised or unsupervised [37]. Supervised models learn from labeled training data (pathogenic/benign) and try to fit the algorithm to give accurate predictions [38]. This type of training dataset is applied for regression and classification problems, so it is the most common in the field of pathogenicity prediction. Unsupervised models, on the other hand, use unlabeled data, and are mainly used for clustering and anomaly detection [39].

**Fig. 2:**
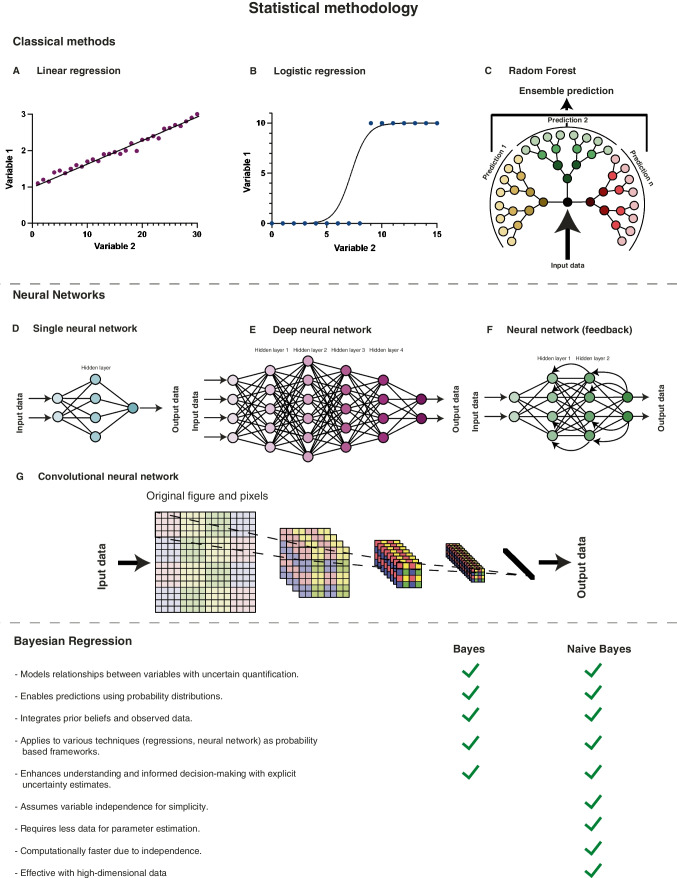
AI statistical models. Classical methods: (**A**) Linear regression describes the relationship between features with a straight line. (**B**) Logistic regression divides features using sigmoidal curves, a more complex approach. (**C**) In random forest, features are consequently divided to improve the classification accuracy. Each decision “tree” starts from a first split in the database called the “root,” and the division continues creating “branches.” The classification output of each “tree” is combined with other “trees,” creating a “forest,” and the most voted option is the output of the model. **Neural Networks**: Neural networks draw inspiration from biological neural networks where nodes (neurons) communicate via connections (axons and dendrites) with weighted synapses, optimizing information flow. In contrast to classical techniques, neural networks can construct planes or hyperplanes (in multiple dimensions) to effectively separate features. (**D**) A basic neural network includes an input layer, up to three hidden layers, and an output layer. (**E**) Deep neural networks can comprise hundreds of layers, enhancing overall performance but requiring complex development. (**F**) Feed-back neural networks allow signals to traverse layers bidirectionally. (**G**) Convolutional networks are primarily employed for image recognition. **Bayes**: This approach models relationships while quantifying uncertainty. The Bayes approach can be applied to classical models and neural networks, enabling predictions with probability distributions, and incorporating prior information of the problem into the model. Bayesian models enable a deeper understanding of the underlying data and facilitate informed decision-making. In contrast to standard Bayesian models, Naïve Bayes assumes independence between features, simplifying models

In terms of the statistical methodology, the primary techniques encompass classical methods, neural networks, and Bayesian regression [35] (Fig. 2). The most basic classical technique is linear regression, where the relationship between one or more numerical features is described using a straight line (Fig. 2A). A more complex classical technique is logistic regression since the relationship is estimated by a sigmoidal curve (Fig. 2B). Decision trees and random forest are also classical techniques. They are trained by supervised datasets and are mostly used for classification and regression. Each “tree” starts from a “root,” the first split in the dataset that best divides the data into their respective classes. After the split, the process can continue creating “branches.” To create a “forest,” the dataset can be divided into subsamples that are used to create multiple “trees,” and the majority vote among them is used as the final model [36] (Fig. 2C). Neural networks are inspired by biological neural networks, where each node (neuron) communicates with others via connections (axons and dendrites), and these connections are weighted to provide an optimized output. In contrast with classical techniques, neural networks can find planes or hyperplanes (more than three dimensions) to separate the features. The most basic neural network consists of an input layer, up to three hidden layers, and an output layer (Fig. 2D). However, deep neural networks can contain hundreds of layers. This structure gives deep neural network a better overall performance, but they are harder to develop (Fig. 2E). In addition to the number of layers, there are multiple types of neural networks, such as feedforward (standard, a layer communicates with the next one), feed-back (signals can go back in layers), or convolutional (mostly used for image recognition) [40] (Fig. 2F and G). Finally, the Bayesian approach is a statistical method that models relationships between variables while quantifying uncertainty, enabling predictions with probability distributions, informed by prior beliefs, and observed data [41]. The Bayesian approach can be applied to other techniques such as classical regressions or neural networks, treating coefficients as probability distributions, and capturing uncertainty in predictions. Bayesian methods give the possibility to incorporate prior information into the model, a probability distribution that represents biological knowledge, or assumptions about the possible values of a parameter before observing any data. Bayesian models enable a deeper understanding of the underlying data and facilitate informed decision-making, in contrast to black-box models that offer predictions without explicit uncertainty estimates. Naïve Bayes is a classification technique that assumes conditional independence between features, making it computationally efficient but potentially oversimplifying real-world relationships (Fig. 2 lower panel).

## Pathogenicity Prediction Software for FH: Analysis of Human Genetic Variants

Single nucleotide variants (SNVs) represent most of the human genetic variants and constitute a major class of genetic risk across common and rare diseases [42]. The SNVs of special interest are those in which an amino acid is substituted, known as non-synonymous SNVs (nsSNV) since they can affect the biological function of a gene product in several ways. In fact, most of the variants that cause FH described so far are missense, ranging from 46%, 52%, and 83% in *LDLR*, *PCSK9,* and *APOB*, respectively [43]. The effect introduced by a missense variant is difficult to predict; in fact, many of the LDLR variants classified as pathogenic by in silico predictions were later reclassified after cascade screening and co-segregation studies [44].

The American College of Medical Genetics and Genomics (ACMG) proposed a specific nomenclature and criteria for the classification of pathogenic variants [45]. This classification recommends the use of five distinct subclasses: pathogenic, likely pathogenic, uncertain significance, likely benign, and benign, with an exceptional class for nonsense and frameshift variants, which are almost always pathogenic [46]. Following the ACMG recommendations, 824 out of 2104 *LDLR*, *APOB*, and *PCSK9* variants found in FH patients (655, 77, and 92*,* respectively) need functional characterization once the nonsense, frameshift, and the characterized ones are discarded [43]. Still, about 40% of all variants need functional evidence to be classified as pathogenic.

Originally, in silico tools were designed to give priority to in vitro characterization of those variants with higher pathogenic probabilities [47]. In recent years, prediction software has greatly improved in accuracy; however, conclusive diagnosis of a variant is still achieved by in vitro characterization or cascade screening. A predictive model considers different characteristics in order to assess the impact of a given variant on protein function, from the evolutionary conservation of an amino acid or nucleotide among homologous sequences to structure analysis [48]. Thus, several prediction software programs have been developed based on the analysis of each particular feature alone or by a combination of several of them.

Prediction software programs can be classified depending on the analyzed parameters (sequence conservation or structural and physicochemical parameters) or the technique used (machine learning and AI) [49]. The latest trend in the development of predictive software involves combining existing models to create innovative frameworks with enhanced accuracy and scope. This approach is revolutionizing the way models are developed, resulting in more robust variant assessment.

Each software has its own weaknesses and strengths, depending on the analyzed criteria, the developed algorithm, and the examined protein. In some cases, a model can be entirely focused on a specific protein, which usually improves the accuracy of the prediction. For these reasons, a prediction is more accurate as there is agreement/consensus among different predictive tools. The ACMG guidelines recommend the use of multiple software packages at once for a more reliable interpretation [45].

There is no guidance/recommendation on which software should be used or how many of them should agree for a prediction to be considered reliable [50•]. In this review, the most commonly used software for predicting the effect of a missense variant in *LDLR*, *APOB*, and *PCSK9* are described, both general and specific ones, to facilitate the selection of the most appropriate for a given variant. Below, the specific features and characteristics of the most commonly used software for predicting *LDLR*, *APOB*, and *PCSK9* missense variants are summarized.

### General Predictive Software

Most predictive software has been trained with a wide variety of protein databases, so they are able to predict the pathogenicity of a broad number of proteins. However, they may fail to diagnose proteins with unique characteristics, or their accuracy may be lower.

#### Early Generation Predictive Software

Several models, including SIFT (Sorting Intolerant From Tolerant), Polyphen-2, and MutationTaster, have paved the way for the advancement of modern predictive tools. These models leverage a variety of features, such as sequence conservation, physicochemical properties of amino acids, and protein structural information, to assess the potential pathogenicity of genetic variants. These software tools were pioneers in the field of pathogenicity prediction and laid the foundation for subsequent developments. Despite their older age, they remain valuable and have been widely used in the scientific community.

##### SIFT

SIFT (https://sift.bii.a-star.edu.sg/) is based on protein sequence homology and conservation to predict the pathogenicity of SNPs (single nucleotide polymorphisms) using Bayes [47]. SIFT classifies the queries as tolerated when the change is predicted to not compromise the protein’s function, or not tolerated, when an alteration is predicted. This software presumes that well conserved amino acids are important, so its prediction relies solely on amino acidic sequence. Therefore, SIFT can evaluate missense variants only when homologous sequences are available, and it is especially suited for sequences with well-aligned orthologous sequences [51].

##### PolyPhen-2

Polymorphism Phenotyping v2* (*PolyPhen-2) (http://genetics.bwh.harvard.edu/pph2/), one of the most widely used pathogenicity predicting software, uses both protein sequence- and structure-based features to evaluate variants, and the effect is predicted by a Naïve Bayesian classifier [52]. The sequence-based features include position-specific independent counts (PSIC) [53] scores and multiple sequence alignment (MSA) [54] properties, and the position of variants in relation to domain limits. The structure-derived features are solvent accessibility, changes in solvent accessibility for buried residues, and crystallographic B-factor [48].

##### MutationTaster

MutationTaster (https://www.mutationtaster.org/) is a software that integrates information from different biomedical databases and analyses evolutionary conservation, splice-site changes, loss of protein features, and changes that might affect the amount of mRNA by a Naïve Bayesian classifier [55]. MutationTaster contains data of SNPs and deletions from 1000 Genomes Project [56] and pathogenic variants found in ClinVar and Human Gene Mutation Database (HGMD) [57]. Common variants in the 1000 Genomes Project are automatically classified as benign, while variants annotated as pathogenic in ClinVar are automatically categorized as pathogenic. Although developed in 2014, a new version of MutationTaster implementing random forest was released in 2021.

#### Recent Generation Predictive Software

Recognizing the necessity for enhanced accuracy and broader coverage, more recent pathogenicity prediction models have emerged, further building upon the predictions of their predecessors. The latest trend in AI is ensemble models. Ensemble models, an innovative approach in predictive analytics, combine the outputs of multiple individual models to bolster accuracy and robustness in predictions. This technique’s advantage lies in minimizing individual model limitations while capitalizing on their strengths, yielding more reliable outcomes. The guiding principle in designing ensemble methods has been “many heads are better than one” [58]. There are many ways of combining and weighting the “base” models, such as bagging (different data samples per model), boosting (sequential training), or stacking (same data samples for each model followed by a meta-model). Several models have been created with this approach in recent years, such as MetaLR and MetaSVM [59], Eigen [60], DANN [61], Condel [62], etc. In this review, we will focus on CADD (Combined Annotation-Dependent Depletion), REVEL (Rare Exome Variant Ensemble Learner), and Varity, some of the most accurate models.

The development and training of these models is much more complex, as they incorporate a larger number of features, and their training is more complex, often involving AI and larger datasets. However, the larger the amount of data available, the more accurate the model will be. The sum of these three factors (larger databases, models to rely on, and the power of AI) has led to the development of predictive models with an accuracy never seen before.

##### REVEL

REVEL (https://sites.google.com/site/revelgenomics/) is an ensemble method for predicting the pathogenicity of missense variants on the basis of combining many individual tools focused on rare variants [63]. REVEL uses bagging random forest technique to incorporate 18 pathogenicity scores from 13 prediction tools (MutPred, FATHMM v2.3, VEST 3.0, PolyPhen-2, SIFT, PROVEAN, MutationAssessor, MutationTaster, LRT, GERP +  + , SiPhy, phyloP, and phastCons). The score depends on the preference of the user since the threshold can be modified to improve the sensitivity or the specificity. REVEL was suggested as the optimal in silico predictor by ClinGen [64] FH variant curation expert panel in the guidelines for *LDLR* variant classification [65].

The REVEL method has strengths in several dimensions. It was trained and evaluated on recently discovered disease-causing and neutral variants, similar to possible future variants found in NGS studies. REVEL’s integration of a diverse set of predictors enriches its predictive power. In addition, REVEL’s careful exclusion of training variants from its predictor components reduces overfitting problems. Demonstrating very high overall performance in independent evaluations, REVEL particularly excels in discerning disease-causing from rare neutral variants. Its value lies in prioritizing relevant variants amid the wealth of rare findings in sequencing analyses, facilitated by pre-calculated scores available for access. The use of the method extends to case–control studies at the genetic level, as evidenced by its adoption in the International Prostate Cancer Genetics Consortium or ClinGen. Its applicability to genes could be studied in the future to interpret variants of unknown significance in various clinical conditions.

##### CADD

CADD (https://cadd.gs.washington.edu/) gives pathogenicity scores based on diverse genomic features derived from surrounding sequence context, gene model annotations, evolutionary constraint, epigenetic measurements, and functional predictions [66]. CADD considers the pathogenicity scores of SIFT or PolyPhen-2 but also takes into account models such as mirSVR [67] (ranks microRNA target sites) or Genomic Evolutionary Rate Profiling (GERP) [68] (evolutionary constraints). The model uses logistic regression to fit the data. A differential feature of this software is that CADD is not trained on characterized genomic variants; it uses less biased, much larger training sets. Another unique aspect of CADD is that it does not specify a cut-off value for pathogenicity scores above which a variant is declared pathogenic or benign.

##### Varity

Varity (http://varity.varianteffect.org/) is a model optimized to detect rare variants [69]. It is based on four feature types: conservation, physicochemical properties, protein–protein interaction, and structure-related properties. It considers more than 30 parameters, scores from pathogenicity predictive tools such as SIFT, PROVEAN [70] or Evm [71] among others, and uses random forest and logistic regressions to optimize the output. The model was specifically trained with rare (minor allele frequency [MAF] between 0.5 and 10^–6^) and extremely rare (MAF < 10^–6^) missense variants from ClinVar, although it is still very accurate with common variants. Being the most recent one, it was tested against CADD and REVEL, outperforming them.

**Fig. 3:**
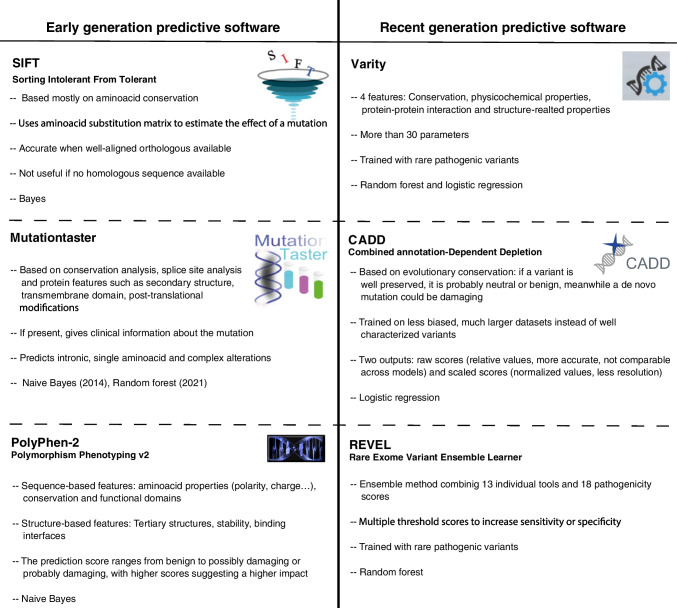
**Pathogenicity Prediction Software**. Early generation: Based mostly on conservation, physicochemical properties, and structure. SIFT predicts pathogenicity of SNPs based on protein sequence homology based on the Bayes approach. It focuses on well-conserved amino acids and requires homologous sequences for accurate evaluation of missense variants. PolyPhen-2 utilizes both protein sequence- and structure-based features to evaluate variants using Naïve Bayes. It incorporates position-specific independent counts (PSIC) scores, multiple sequence alignment (MSA) properties, and structural characteristics to predict the effect of variants. MutationTaster integrates information from biomedical databases to analyze evolutionary conservation, splice-site changes, loss of protein features, and mRNA-related changes for predicting pathogenicity. It uses a Naïve Bayesian classifier. **Recent generation**: These modern models use ensemble techniques that combine the output of multiple individual models to get a more precise result. REVEL uses random forest to integrate 13 prediction tools, focused on rare missense variants. It was trained with recently discovered variants to mimic possible variants found in the future. CADD provides pathogenicity scores based on diverse genomic features and considers pathogenicity scores of SIFT and Polyphen-2 among others. It employs large training sets and logistic regression to fit the data. CADD does not rely on a specific cut-off value for pathogenicity determination. Varity is based on 4 feature types: conservation, physicochemical properties, protein–protein interactions, and structure-related properties. It considers more than 30 parameters and uses random forest and logistic curve to optimize the output. Varity also focuses on rare missense variants

The information provided above is illustrated in Fig. 3.

### Predictive Models for LDLR Genetic Variants

In recent years, there has been a rapid development of predictive software for specific proteins, which has provided very specialized models capable of considering specific nuances of each protein. The development of a model of this type requires the existence of extensive databases containing many variants of the protein to be studied. In this way, and by applying machine learning models, the software can be primed with enough information so that it can provide accurate results. Due to the relatively high frequency of nonsense variants of *LDLR*, it has been possible to generate extensive databases (ClinVar, LOVD) that allow the development of highly accurate predictive models.

## SFIP-MutID

Structure-based Functional Impact Prediction for Mutation Identification* (*SFIP-MutID) was developed using structural models of LDLR at both neutral and acidic pH [72]. The structures of LDLR were obtained from the protein data bank (PDB ID, 1F5Y, 1N7D), and to visualize the effect of each variant, homology modeling in Discovery Studio [73] was used. SFIP-MutID considers three aspects of the protein: the affected domain (variants in some domains are more likely to be pathogenic), the structure of the affected area, and, an energy-based score that classifies variants as destabilizing or not. However, this model does not cover certain regions of the LDLR because the structure of the protein is not completely resolved [72].

## MLb-LDLr

The most recent LDLR-specific predictive software is Machine Learning-based LDLr (MLb-LDLr) [74]. This model is trained on the ClinVar database, where more than 1300 *LDLR* missense characterized variants are annotated (last update: November 2022). It considers seven features of the altered amino acid to give a prediction: conservation of the substituted residue, original and substituting amino acids, charge, hydrophobicity, size change, and affected domain. Mlb-LDLr is an open-access predictive software provided to the scientific community (https://www.ehu.eus/en/web/hypercholesterolemia-mechanisms/mlb-ldlr1). The introduction of a machine learning algorithm provides a predictive model with a specificity of 92.5% and a sensitivity of 91.6%, which shows high accuracy in predicting both pathogenic and benign variants.

## Predictive Models for APOB Genetic Variants

Compared to the large study carried out in *LDLR*, *APOB* genetic variants are not as well characterized, and pathogenicity predictions for this protein are much less reliable. While most proteins pathogenic variants tend to occur in highly conserved regions, *APOB* variants are distributed all over the protein [75]. In fact, the most used in silico tools have failed to correctly predict some of the most common *APOB* variants [76], except for genetic variants with a major involvement in receptor binding [20–22]. These results indicate that computer-based predictions of functional effects cannot yet reliably predict the effect of *APOB* SNVs. Several factors underlie the inaccuracy in the prediction of the effects of a missense apoB-100 variant, among them: the huge size of the protein, the lack of a crystallographic structure of the native protein, and the nature of protein folding within a lipid moiety, which cannot be addressed by in silico tools to study the protein–lipid interactions.

In addition to the pathogenic receptor binding variants of apoB-100, there exist other pathogenic variants that exert an impact on the structural integrity of the apoB molecule, leading to the impairment of very low-density lipoprotein (VLDL) and low-density lipoprotein (LDL) assembly processes. These genetic variants underlie the etiology of hereditary familial hypobetalipoproteinemia (FHBL), a clinical condition distinguished by compromised hepatic lipid secretion and limited transport to peripheral tissues. The spectrum of *APOB* pathogenic variants contributing to both biallelic APOB-FHBL and heterozygous APOB-FHBL is largely represented by frameshift, nonsense, and splice variants. These variants result in the production of a truncated apoB protein, characterized by an incomplete sequence, thereby perturbing its functional properties. Consequently, this perturbation results in marked reduction in levels of total cholesterol, LDL,VLDL, and serum triglycerides [77].

In this context, precise discrimination between variants that elevate and those that lower LDL cholesterol levels is crucial for clinical accuracy. Prediction software must effectively distinguish such pathogenic variants, holding paramount significance in guiding appropriate treatment strategies and understanding patient well-being implications. Given the multifaceted role of apoB-100, these considerations are pivotal for ensuring clinical management and genetic interpretation precision.

## Predictive models for PCSK9 genetic variants

Predicting the functional consequences of missense variants in PCSK9 is a challenging task due to the varied outcomes they can produce. While some variants result in reduced PCSK9 function and are deemed benign, others lead to increased activity, thereby causing autosomal dominant hypercholesterolemia. Consequently, the prediction of PCSK9 pathogenicity introduces additional complexity as software tools must not only anticipate deleterious effects on protein structure or conformation but also determine the variant’s pathogenic, atheroprotective, or neutral nature.

Assessment of the performance of software tools such as SIFT and PolyPhen-2 in analyzing GOF and LOF variants in multiple genes has been conducted. The findings indicate that both software tools exhibit heightened sensitivity and specificity for LOF variants compared to GOF variants [78]. This behavior can be attributed to the fact that LOF variants often involve substantial amino acid substitutions with significant physicochemical changes, thereby instilling greater confidence in the predictions. Moreover, GOF variants are less prevalent, resulting in less extensive algorithm training. When applying this understanding to PCSK9, one can anticipate higher accuracy in predicting LOF variants, despite the existence of a greater number of reported PCSK9 GOF variants. Nonetheless, certain regions of the PCSK9 protein, such as the LDLR binding site and furin cleavage site [28], are well characterized, allowing for more accurate in silico predictions for variants occurring in these areas.

This highlights the intricate nature of prediction within the complex landscape of PCSK9’s functionality. Consequently, critical importance lies in the capacity of prediction software to accurately distinguish between GOF pathogenic variants and LOF cardioprotective variants. It becomes increasingly evident that the mere labeling of a PCSK9 variant as “pathogenic” by software does not guarantee its attribution as a cause of FH. This is particularly relevant as software might interpret a LOF variant as pathogenic due to its failure to meet the criteria for benign classification, even though it may possess beneficial physiological outcomes.

Despite the complexities involved, the performance of commonly used pathogenicity predictive software for *LDLR*, *APOB*, and *PCSK9* can be assessed empirically. Table 1 summarizes the expected performance of these software tools based on the available data.

**Table 1:** Pathogenicity predictive software expected performance on *LDLR*, *APOB*, and *PCSK9*

Pathogenicity predictive models	*LDLR*74	*APOB*	*PCSK9*
SIFT	High accuracy -Sn: 86% -Sp: 88%	Low accuracy -Lack of structure -Low conservation	Regular -Unknown mechanism -LOF > GOF79
PolyPhen-2	High accuracy -Sn: 93% -Sp: 88%	Low accuracy -Lack of structure	Regular -Unknown mechanism -LOF > GOF79
MutationTaster	High accuracy -Sn: 95% -Sp: 78%	Low accuracy -Low conservation	Regular -Unknown mechanism
REVEL	High accuracy* -Sn: 95% -Sp: 90%	Low accuracy -Low conservation -Low performance base models	Regular -Unknown mechanism -Low performance base models
CADD	High accuracy -Sn: 89% -Sp: 94%	Low accuracy -Low conservation	Regular -Unknown mechanism
Varity	High accuracy* -Sn: 94% -Sp: 91%	Low accuracy -Low conservation	Regular -Unknown mechanism -Low performance base models
SFIP-MutID	Regular -Sn: 90% -Sp: 22%	-	-
MLb-LDLr	High accuracy -Sn: 91% -Sp: 91%	-	-
Overall analysis	Reliable predictions	Unreliable predictions	Moderate predictions

*Sn* Sensitivity, *Sp* Specificity.

* Unpublished data currently under review in another study by Larrea-Sebal.

## Structure-Modeling Software

Since the structure of a protein determines its function, protein structure prediction (PSP) is a major challenge in biochemistry. A protein’s ability to fold into different conformations is essential for the viability of many biological processes. Therefore, knowing the 3D conformation of a protein is decisive for being able to predict the effect of a variant on its biological function [80, 81••].

The determination of a protein’s structure has traditionally relied on high-resolution experimental techniques such as X-ray crystallography [63], NMR spectroscopy [64], and cryo-electron microscopy [65]. While these methods yield the most precise protein structures, the process of crystallization poses a significant bottleneck, particularly given the vast number of protein sequences to be solved [66]. This situation highlights the necessity of generating in silico models to provide accurate structure predictions. In this sense, two main PSP approaches have been used over the years: template-based modeling (TBM) and template-free modeling (FM) methods. TBM methods use the structural framework of existing proteins obtained from the PDB, while FM methods predict the structure without any template. The accuracy of TBM relies on the existence of evolutionary similar proteins, obtaining very precise predictions for proteins with high sequence identity (SI) templates. However, when SI drops below 30%, the accuracy of the model decreases [82]. In those cases, FM methods are more useful because they are based on physics- and knowledge-based energy functions. As they construct protein structures from scratch, they are often referred to as ab initio or de novo modeling approaches [79, 83]. In general, FM does not achieve the same accuracy as TBM, but the gap between both methods is narrowing thanks to the use of deep learning approaches [84, 85].

**Fig. 4:**
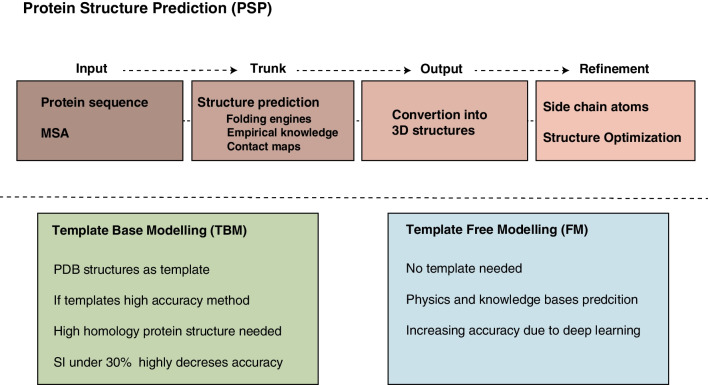
Protein Structure Prediction Workflow. PSP models, comprising input, trunk, output, and refinement modules, enable protein structure prediction. The input, usually the protein’s primary sequence, is analyzed by the trunk component using folding engines, sequence-structure knowledge, or contact maps to predict the structure. The output module converts the trunk-generated structures into 3D structures by determining atomic coordinates. Refinement involves optimizing the conformation and adding side-chain atoms. PSP models focus on predicting structural effects rather than variant pathogenicity. Nevertheless, they provide valuable insights into the impact of variants on protein structure, including intermolecular interactions at binding sites

PSP is generally composed of four main components: the input, a trunk, an output, and a refinement module [86]. The primary input is typically the protein’s primary sequence, although modern models have incorporated additional information such as homologous multiple sequence alignment (MSA). The trunk component analyzes the input data and utilizes folding engines, empirical knowledge of sequence-structure properties [87, 88], or more recently, contact maps (binary matrices encoding residues likely to be in contact) [89, 90] to predict the protein’s structure. The structures generated by the trunk component are subsequently transformed into 3D structures by the output module, which determines the atomic coordinates of the protein. Finally, the resulting 3D structure undergoes refinement, which includes the addition of side-chain atoms and optimization of the overall conformation. Unlike prediction software, the purpose of these models is to forecast the effect on the structure of the protein and not the pathogenicity of a variant. With that information, the effect of the variant can be inferred, either at the structural level or at the intermolecular level, if it affects the binding site with another molecule [91] (Fig. 4).

Although PSP algorithms have been used for a long time, very recently, a breakthrough has occurred with the development of AlphaFold, a new software that predicts the protein structure of an amino acid sequence, and allows visualizing and analyzing the results. AlphaFold is a structure prediction software developed by DeepMind [85] that first appeared in the 13th edition of *Critical Assessment of Structure Prediction* (CASP) [92]. CASP is a community-wide assessment that tests the ability of various software to model protein structure from amino acid sequence [93]. In CASP13, AlphaFold received exceptional ratings, outperforming all other models by a wide margin. Because of this, AlphaFold was considered an anomalous leap in the field of protein structure prediction [94]. AlphaFold is based on co-evolution-dependent methods. These models work by detecting residues that co-evolved, i.e., that have mutated together over time, suggesting that both residues interact and are close to each other in 3D space [95]. Using this approach, it can be inferred whether two residues are in contact or not (binary contact matrices), which allows the acquisition of 3D coordinates. However, AlphaFold uses a more sophisticated co-evolution-dependent method based on RaptorX [96]. Instead of predicting binary contact, Raptor X predicts the distance between residues using discretized spatial ranges and calculates the mean and variance of the predicted distribution to localize each atom. Furthermore, AlphaFold uses a hundred-layer deep learning neural network to solve the structure, a technique never before applied to PSP [97]. All in all, AlphaFold, and its newer version AlphaFold 2 released for CASP14 (2020), are the most accurate PSP models available [98].

In addition to structure prediction, recent PSP algorithms also include the ability to predict the effect of missense variants using structure analysis [91, 99–109]. These algorithms predict 3D protein structure from amino acid sequences by searching for the most stable 3D state under native conditions. If a pathogenic variant destabilizes the molecule, then it can be expected that the algorithm will predict a more unfolded state [110]. However, these models are still in their very early stages of development and are not very accurate. For example, the predictive capability of AlphaFold-2 was tested on 2648 single-point missense variants over 121 proteins, with accurate results when compared to experimentally obtained structures [91]. Nevertheless, other studies reported that while AlphaFold is currently unable to predict structural effects of missense variants, it is conceivable that incorporation of experimental data and a database for storing structure-disrupting pathogenic variants will enable this feature in future versions of protein structure prediction programs [110, 111].

PSP software can be especially useful to study the effect of a missense variant on FH-related gene variants since many of the pathogenic variants occur at intermolecular interaction sites. ApoB-100 interacts with LDLR to be internalized, and the interaction of PCSK9 with LDLR regulates receptor expression. The residues involved in both processes are well characterized, and due to the lack of the complete structure of LDLR and apoB-100, PSP software can be decisive.

### Structure-Modeling of LDLR

LDLR is a well characterized protein, with tens of X-ray crystallographic and NMR solved structure-files available on Uniprot. However, none of them covers the entire protein, with most of them covering a few hundred residues with X-ray resolution ranging from 1.2 to 7 Å. The most complete solved structure covers the ecto-domain, amino acids 22–720 [112] (PDB ID 1N7D). There are crystals of both LDLR alone and LDLR with stabilizers such as PCSK9. The prediction made by AlphaFold is also available, but there are some discrepancies between the AlphaFold predicted structure and the crystal structure. LDLR requires calcium ions to enable correct folding of the protein’s ligand binding domain and ligand binding [113]. Without calcium, LDLR is not functional, so crystallographic experiments have been carried out in the presence of calcium. However, AlphaFold does not consider this parameter, so the obtained prediction is not accurate. Despite this, AlphaFold can be used for the structural prediction of other domains not affected by calcium ions, such as the β-propeller or the EGF domains; however, most of the structures obtained from X-ray crystallography do not cover the LDLR C-terminal sequence (400–860 residues).

### Structure-Modeling of apoB-100

Being part of a lipoprotein, apoB-100 interacts closely with lipids and remains with the lipoprotein throughout its metabolism [114]. ApoB-100 is highly insoluble in aqueous solution, and due to its nature, it has not been possible to obtain its structure by crystallography [115] or electron cryo-microscopy [116]. Solving the apoB-100 structure using PSP has not been successful either, and nowadays, the only predicted structure available on Uniprot (P04114) was obtained by SWISS-MODEL[117]. However, this PSP is not accurate, and does not resemble the belt-like structure that surrounds LDL particles.

### Structure-Modeling of PCSK9

The structure of PCSK9 is well known, with plenty of PCSK9 structures solved by X-ray crystallography on Uniprot [118–120]. Most of the structures are accurate, covering the entire protein with a resolution of 2 to 3 Å. PCSK9 crystals have been obtained with and without adjuvants, sometimes even in complex with LDLR, which helps understanding how PCSK9 interacts with the receptor [121]. Structure prediction made by AlphaFold (PDB ID AF-Q8NBP7-F1) is very accurate, resembling other PCSK9 crystallographic structures (PDB ID 6U3X, 6U2P). Accordingly, this makes it possible to use AlphaFold for predicting the structure of PCSK9 variants, allowing a more in-depth analysis of the variant’s effect. In addition, by combining the extensive knowledge of the binding sites of LDLR and PCSK9, the crystallographic data allows obtaining accurate models to study the effect of a missense variant on the LDLR-PCSK9 complex formation.

## Docking

Protein docking is the process of calculating the 3D structure of a protein complex starting from the individual structures of each protein [122]. It can be considered a further step in obtaining the structure of a protein complex since it also predicts how proteins interact. Molecular docking is a highly used technique in drug design, where it helps predict how ligands may bind to target proteins in 3D. However, protein–protein docking is not widely used [123] and is very helpful to delineate the interaction characteristics, the effect of a variant in the interaction, or affinity predictions [124••]. Intermolecular interactions are pivotal for many biological processes. These interactions are very specific, and any variant affecting the interaction interfaces is more harmful than others [125]. As it happens with the structure of a protein, early docking analysis relies on X-ray crystallography, NMR, and cryo-EM to study the interactions.

Since docking is based on structure, similarly to PSP models, the same two approaches can be taken to obtain the structures: “free” docking and template-based docking [126]. Template-based methods usually yield more accurate predictions when good templates are available, while “free” docking is advised when no template is available. However, docking techniques are also classified depending on the flexibility of the interacting proteins. Rigid-body techniques assume that proteins maintain their structure when interacting with other molecules, while flexible docking considers atoms’ vibration, giving generally more accurate results [127].

The protein–protein docking process consists of two major steps: pose generation and scoring. Pose generation constitutes the first phase and serves to discard near-native structures, which is crucial for an accurate prediction. The native stage of both proteins is obtained by translating and rotating them until a few fitting poses are obtained. The scoring phase involves assigning scores to each possible conformation based on up to five characteristics, depending on the model. Force-field based scores consider non-bonded terms (van der Waals potential) and bonded terms (bond angle) [128, 129]. Empirical scores utilize intermolecular interactions and changes in accessible surface area [130]. The knowledge-based score takes into account existing knowledge on protein interaction [131]. The last two scores are consensus-based [132] and machine learning-based [133].

The performance of docking methods is tested in the blind prediction challenge known as the Critical Assessment of Prediction of Interactions (CAPRI) [134], and it was designed on the model of CASP [135]. Both are community-wide experiments where different predictive models are tested, but whereas in CASP, protein folding is predicted from amino acid sequences, in CAPRI, protein assemblies are modeled by docking component structure [123]. Regarding CAPRI, both free and template-based docking models are tested, and the best scoring models are considered the most accurate ones. In one of the latest calls (2016) [136], the best models were SwarmDock [137], followed by ZDOCK [138], pyDock [139], HADDOCK [140], and Cluspro [141]. These models cover most docking methods; therefore, they are suitable for various types of docking problems.

### Docking Software

#### ZDOCK

ZDOCK is a template-free rigid-body protein–protein docking program that uses a Fast Fourier Transform (FFT) algorithm that considers shape complementarity, electrostatics, and statistical potential for scoring. ZDOCK has been improved by adding the rescoring scheme called IRaPPa (Integrative Ranking of Protein–Protein Assemblies) that, briefly, uses physicochemical descriptors and combines a large selection of metrics to improve the selection of near-native solutions [133, 142]. The portal for running the program M-ZDOCK is available (https://zlab.umassmed.edu/m-zdock/) [143].

#### pyDOCK

Similarly to ZDOCK, pyDOCK is a template-free rigid-body docking program that uses FFT, but its main scoring functions are desolvation and electrostatics. PyDOCK has also been improved by IRaPPa, and it uses ZDOCK for the pose generation, meaning that both software results are similar. The web server for pyDOCKWEB is also freely available (https://life.bsc.es/pid/pydockweb) [144].

#### SwarmDock

SwarmDock, unlike ZDOCK and pyDOCK, is a flexible docking method that utilizes a particle swarm optimization of 350 parameters to optimize the posing [145]. SwarmDock considers 17 structural parameters to optimize the conformation and relative position of each particle. IRaPPa has also been applied to this method.

#### HADDOCK

HADDOCK (High Ambiguity Driven DOCKing) is a semi-flexible docking protocol that uses empirical and bioinformatic scores to drive docking, especially van der Waals and Coulomb electrostatic energies. HADDOCK 2.4 is the software developed with this model, and it is among the most used ones [146].

#### Cluspro

Cluspro is a rigid-body docking method that relies on PIPER for pose generation, a docking program based on FFT [147]. Cluspro differs from other rigid body-based methods because, instead of using the lowest energy structure, it analyzes a cluster of the 1000 lowest ones, assuming that the real docked conformation is not necessarily the one with the lowest energy, but it will probably be in that cluster. Cluspro Web is also widely used software (https://cluspro.bu.edu/login.php) [141].

### Use of Docking to Study LDLR-apoB-100 and LDLR-PCSK9 Interactions

Docking studies can be very helpful when predicting the effect of FH-related variants due to the receptor–ligand nature of LDLR with apoB-100 and PCSK9. A large number of these variants occur on the binding sites of these proteins, which could affect the affinity of the binding and cause FH. Regarding the most appropriate docking software to be used in each case, for LDLR-apoB-100 interaction, as no reliable apoB-100 structure is available, free docking software is advised. On the other hand, for assessing LDLR-PCSK9 interaction, as both PCSK9 and LDLR have well-characterized crystals, template-based software can be used.

#### LDLR-apoB-100 Interaction

The most LDLR affected domains by missense variants are the LBD and EGF-like domains, both key players in binding to apoB-100. Although these regions are highly conserved, not all the pathogenic variants located within these domains are correctly classified by predictive software. Subtle changes in the structure of the protein may lead to a failed prediction by the PSP software. In these cases, docking studies give an in-depth analysis of the effect of the variant on the binding of LDLR and apoB-100, considering not only the LDLR structure but also the interaction with apoB-100.

Docking software has previously been used as an approach to predict the effect of missense variants on the efficiency of binding to apoB-100 and describe the mechanism by which these LDLR variants cause FH [148]. Barbosa et al. characterized six LDLR variants using, among other techniques, docking models to assess the effect of the variant on the binding of LDLR to apoB-100 [149]. LDLR and apoB-100 sequences were obtained from Uniprot, the structure was generated by AlphaFold2, and the protein–protein docking was analyzed following a previously described protocol [148]. Briefly, a cluster of Cluspro, FireDock [150], Haddock, and Patchdock [151] software was used, and the results were ranked according to FFT score values. Overall, docking and molecular interactions analyses showed that p.(Cys184Tyr) and p.(Gly373Asp) LDLR variants alter the interaction of the receptor with apoB-100 [149].

Docking assays could also be performed the other way around, by analyzing the effect of apoB-100 variants on its binding to LDLR.

#### LDLR-PCSK9 Interaction

In the past few years, reliability of PCSK9-LDLR docking-based predictions has largely improved due to the increased number of described GOF- and LOF-PCSK9 variants [152–160] and the availability of several high-resolution crystallographically resolved PCSK9 structures. The structure of the PCSK9-LDLR complex has also been resolved, thus making the use of docking highly recommended to study the nature of their interaction. As shown in recent studies, docking can help to understand the mechanisms leading to pathological or beneficial effects. For example, docking has given new insights regarding the mechanisms of action of Ser127Arg [161] and Asp374Tyr [162] GOF PCSK9 variants [163]. These docking studies have indicated that the two pathogenic variants confer significantly higher binding affinity for LDLR as well as different binding modes, which impair LDLR from adapting its closed conformation.

## AI-Driven Enhancement of Predictive Models in Bioinformatics

The integration of AI in bioinformatics has emerged as a pivotal solution to address the challenges posed by the overwhelming amount of biological data. AI techniques, such as machine learning and deep learning, have shown significant potential for handling and analyzing vast datasets, thereby enhancing the accuracy of predictive models. These AI-driven approaches enable researchers to extract meaningful patterns and relationships from diverse biological data sources, including genomics, proteomics, and transcriptomics. By leveraging AI algorithms, bioinformatics researchers are able to develop more sophisticated prediction models that account for intricate interactions within biological systems.

The use of AI has drastically increased in clinical genomics. It has been applied in a wide range of conditions and approaches, such as patient photography analysis (facial analysis for disease identification, radiologic studies, microscopy data) [164], cardiology predictions (hypertension incident, atrial fibrillation, aortic stenosis) [165] blood biomarkers (mantle cell lymphoma [166], anemia [167]), interpretation of copy number variants [168], or classification of non-coding variants [169]. Regarding variant pathogenicity predictions, AI has revolutionized the field, providing advanced tools for accurate assessment. Starting from linear regressions to present-day deep learning models, the performance of these tools has increased exponentially. NGS technologies, platforms such as ClinVar, and advanced high-resolution crystallography techniques have led to the creation of extensive databases, enabling the development of highly precise predictive models. Models often have access to the position where the genetic variant occurs, evolutionary conservation of the position, prevalence of the variant in the population, probable effect of the variant on the mRNA or protein, affected domains, clinical phenotypes of the individuals, previously seen effects in other patients, family history, etc. AI can process and combine all this information, and the interpretation and weighing of each variable is the key to success.

## Future Perspectives and Conclusions

The rapid development of bioinformatics tools has dramatically increased the accuracy of prediction software, both in assigning pathogenicity values to variants and in predicting their effect on the structure of the protein. Even so, there is still room for improvement, especially in obtaining protein structures and predicting protein–protein interactions. Most prediction algorithms are trained on empirical results, such as structures obtained by X-ray crystallography or functional in vitro characterization of variants, so the sensitivity and accuracy of in silico predictive tools are limited by the amount of available experimental data.

Algorithms can also be improved from within by applying deep learning techniques [170]. Deep learning has already been implemented in some PSP processes such as MSA, contact map prediction, or convolutional neural networks, and AlphaFold and RaptorX are based on these advanced machine learning tools. Regarding pathogenicity prediction models, the combination of ensemble techniques and deep learning has emerged in recent years. Until recently, ensemble and deep learning models were regarded as separate methods in bioinformatics. Nowadays, the blending of these two popular techniques is causing a new wave of progress and the use of next-gen machine learning methods, known as ensemble deep learning [171, 172]. The next big breakthrough in the predictive software field will arise from ensemble deep learning implementation in every aspect of the algorithms.

Considering the bioinformatic tools mentioned in this review and their performance on the three most frequently FH-involved genes, different approaches should be taken to predict the pathogenicity of a variant. Pathogenicity prediction software is very accurate for *LDLR* variants, showing a very high hit rate when analyzing variants submitted in ClinVar, so this software should be the first approach to inferring the effect of an *LDLR* variant. Then, depending on the location of the genetic variant, PSP and docking software can be used to analyze the possible effect on the biological function of the receptor. The 3D structure of the ectodomain is well characterized, but there is only one submission of the intracellular domain (778–860 residues) in Uniprot, and it does not cover the entire region. Finally, docking software should be considered for genetic variants occurring in the ectodomain, especially those affecting LBD or EGF-like domains, which interact with ligands, apoB-100 and PCSK9.

As for *APOB*, bioinformatic predictions seem to not be very accurate yet. It is discussed that this may be due to its interaction with lipids and its polymorphism, which affect both the conservation of residues and the quaternary structure of the protein. These two parameters, conservation and structure, are key factors for both pathogenicity prediction and PSP software, which hampers the usefulness of predictive software. Even with these drawbacks, bioinformatic tools have been used for *APOB* variant pathogenicity prediction and protein–protein docking assays with LDLR [149]; therefore, they should not be dismissed. Accurate discrimination between LDL cholesterol-raising and lowering variants is essential for future clinical precision. Effective differentiation in prediction software is crucial for guiding treatments and understanding patient health implications, especially given the complex role of apoB-100.

**Fig. 5:**
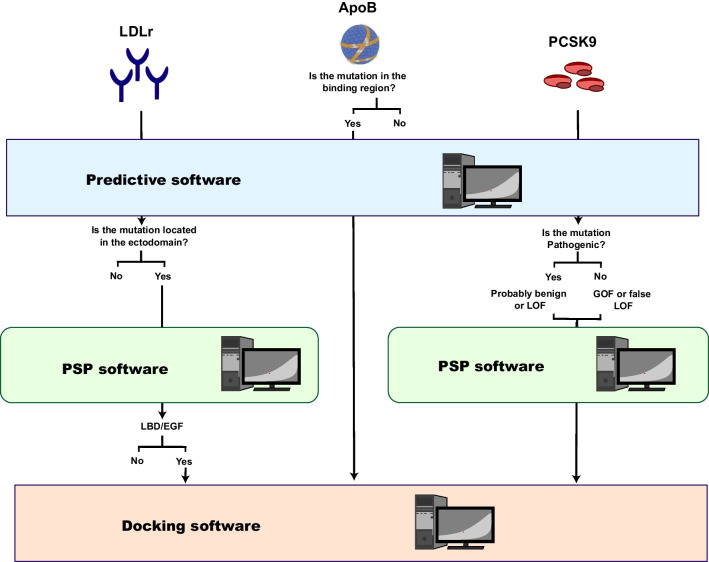
Workflow illustrating the selection of bioinformatics tools based on the mutated gene and affected domain for accurate pathogenicity prediction. Predictive software demonstrates high reliability in assessing genetic variants within the *LDLR* gene. Specifically, when the variant occurs in the ectodomain, it is recommended to utilize PSP software. Conversely, if the variant affects the ligand-binding domain (LBD) or epidermal growth factor (EGF) domains, the analysis should be complemented with docking software for comprehensive evaluation. In the case of *APOB* variants, pathogenicity predictions can be considered reliable solely when the variants impact the binding domain. In such instances, the integration of docking software can further enhance the analysis. However, it is important to note that pathogenicity predictions for *APOB* may be limited in other regions, requiring additional approaches for accurate assessment. Regarding *PCSK9* pathogenicity predictions, they provide valuable insights but may not offer a comprehensive diagnostic assessment of the variant. To obtain a more in-depth analysis, the combined utilization of predictive software and docking software is recommended. This integrated approach allows for a thorough investigation of PCSK9 variants and their potential implications

Regarding PCSK9, the structure of the protein is well described, and crystals covering the entire protein are available in Uniprot. However, due to the GOF or LOF variants, software prediction is not accurate. PSP software can accurately predict the effect of a variant on the 3D structure, but the interpretation of the results remains in the hands of the researcher. In any case, the use of this software reveals whether the variant affects the protein structure in any way, and if it does, this variant should be a candidate for in vitro characterization. The importance of future directions becomes evident in the vital task of software accurately distinguishing between GOF pathogenic variants and LOF variants. On the other hand, docking studies are more reliable, especially if the variant occurs in a binding region, since the direct interaction is analyzed. A full workflow of the software that should be used for each gene variant is shown in Fig. 5.

In conclusion, the integration of bioinformatics tools, protein structure modeling, and docking methodologies offers great promise for advancing our understanding of FH. These approaches provide valuable insights into the pathogenicity of genetic variants, protein structure–function relationships, and intermolecular interactions. Future advancements in AI and these methodologies hold the potential to enhance FH diagnosis, risk assessment, and the development of personalized treatment strategies for affected individuals.
